# Process Mapping to Support the Implementation of a Regional Strategy to Address the Opioid Epidemic

**DOI:** 10.3390/healthcare12191995

**Published:** 2024-10-06

**Authors:** Yifei Liu, Stacy L. Farr, John A. Spertus, Danielle M. Olds, Tracey A. LaPierre, Holly N. Renwick Hagle

**Affiliations:** 1 University of Missouri—Kanas City Healthcare Institute for Innovations in Quality, Kansas City, MO 64108, USA; 2 Division of Pharmacy Practice and Administration, University of Missouri—Kanas City School of Pharmacy, Kansas City, MO 64108, USA; 3 Saint Luke’s Health System, Kansas City, MO 64111, USA; 4 Department of Biomedical and Health Informatics, University of Missouri—Kanas City School of Medicine, Kansas City, MO 64108, USA; 5 Department of Sociology, University of Kansas, Lawrence, KS 66044, USA; 6 Collaborative to Advance Health Services, University of Missouri—Kanas City School of Nursing and Health Studies, Kansas City, MO 64108, USA

**Keywords:** opioid epidemic, process map, peer recovery coach, implementation

## Abstract

Background/Objective: To address the opioid epidemic in Kansas City, Missouri, local health systems sought to implement a referral to peer recovery coaches (PRCs) for clients presenting with opioid use disorder. Client referrals were made primarily through health system emergency departments, where PRCs met clients to facilitate linkages to recovery support for up to twelve months. This study aimed to evaluate and improve program implementation with process mapping at three local health systems. Methods: Using a five-phase conceptual framework and three development and implementation domains, providers, administrators, and PRCs were interviewed to identify the process for recognizing clients with opioid use disorders and referring them to PRCs. Serial meetings were held to validate the process maps at three health systems and a distillation of key processes was created to guide future analyses and implementation efforts. Results: A detailed process map for each health system was developed, from which a high-level process map was created to support future implementation efforts. Health system-specific process maps varied, although conceptually coherent elements were identified across each system to diagram a recovery ecosystem to support client referrals to PRCs. Conclusions: By systematically assessing the implementation of the same program across different health systems, critical steps, along with their barriers and facilitators, were identified that can be used to understand the processes of care associated with outcomes and to guide future implementation efforts.

## 1. Introduction

While opioid use disorders are a national epidemic in the U.S., there is a geographic variation in patient encounters with opioid use disorders, both nationally and in the Midwest [1,2]. The State of Missouri, a Midwestern state, has experienced a disproportionate burden, with an overdose death rate exceeding the national average (27.1 vs. 24.7 per 100,000 population) [3]. To help address this challenge, the Missouri Hospital Association created the Engaging Patients in Care Coordination (EPICC) program in 2016 in the Saint Louis area to connect clients with substance use treatment and recovery resources using peer recovery coaches (PRCs) [4]. The EPICC program is based on a risk-reduction and recovery-oriented care model that includes referrals from participating health systems, primarily through their emergency departments (EDs). PRCs are people with lived experience who have been in recovery for at least two consecutive years and have received training certification for their role from the State of Missouri’s Department of Mental Health. The PRCs meet clients on site to facilitate immediate linkages to recovery support services that include evidence-based treatment for opioid use disorder and community-level care coordination. Each client engagement was designed to include up to 12 months of ongoing support from the same PRC. The EPICC program operates under the guidance of a steering committee which plays a central role in shaping the strategic vision, design, implementation, and evaluation of the program [5]. Committee members, who represent a wide range of expertise and experience, meet monthly to monitor the program’s implementation and progress.

The Saint Louis initiative resulted in 149 client referrals through EDs from December 2016 to June 2017. The results of the Saint Louis initiative provided insights for sustaining and improving the EPICC program to serve a larger population of opioid overdose survivors, strengthen community connections, and facilitate increased access to healthcare professionals. Following the initiative’s success in the eastern region of Missouri, the Missouri Hospital Association sought to expand the EPICC program to additional health systems in the central, southwestern, and western regions of Missouri in 2019 [4]. The Western Region EPICC (WR EPICC) program was implemented in Kansas City, Missouri.

To better understand the variability of success in the WR EPICC program referrals and ultimately ensure more successful client recovery, we sought to compare and contrast different implementation strategies and processes of care across three participating health systems in Kansas City through qualitative interviews and formal process mapping. These three health systems were denoted as Health System A (consisting of two hospitals), Health System B, and Health System C in this study. By providing a clear and comprehensive overview, process mapping enables a common understanding among collaborators to delineate key milestones, decision points, and actions required at each step, facilitating future implementation efforts. This study had two objectives: (1) to develop a detailed process map for three health systems; and (2) to create a high-level process map summarizing insights across the different implementation efforts.

## 2. Methods

### 2.1. Overview of the WR EPICC Program

In the WR EPICC program, PRCs are employed by a local behavioral health agency, CommCARE, which is responsible for hiring, training, scheduling, and dispatching PRCs to health systems upon referral. CommCARE enrolls clients and manages the referrals for recovery support. Engagement is tailored to each client’s needs and could include peer supports, mutual aid support groups, housing, sober living environments, formal treatment (outpatient or inpatient), pharmacotherapy, and overdose education. The WR EPICC program was offered to multiple health systems in the Kansas City metro area. The program involves multiple steps, collaborators (e.g., healthcare professionals, organizational administrators, and PRCs), and interactions. Accordingly, documenting and customizing the implementation to each health system was deemed critical to improve implementation outcomes and optimize the program’s impact. Because no such efforts had been previously performed, we retrospectively evaluated how each individual health system sought to implement their participation in the WR EPICC program.

### 2.2. Mapping the Process of Implementation

Applying dissemination and implementation (D&I) science methodologies to regional quality improvement activities serves to improve the process of translating evidence-based interventions into real world practice [6,7]. There are five core D&I domains: context assessment and intervention selection, dissemination, adaptation, implementation, and sustainability [8]. Process mapping, defined as the “entire approach that leads to a holistic understanding of the process under review” [9,10], recognizes the complexity of implementing interventions and can be applied across these domains. For example, in the domain of context assessment and intervention selection, process mapping can visually represent the relationships among collaborators, resources, organizational structures, and cultural norms. Through this lens, targeted strategies and interventions can be tailored to specific contexts. In the domain of dissemination, process mapping can identify potential adopters and key decision-makers. Within the adaptation domain, process mapping can reveal which elements are central to intervention effectiveness and which can be changed without compromising effectiveness. In the domain of implementation, process mapping can capture the interactions among various collaborators, helping to identify potential barriers and facilitators, as well as opportunities for process efficiency improvement, collaboration, and coordination. Lastly, within the sustainability domain, process mapping can highlight the activities required for long-term success, so collaborators can proactively address challenges and allocate resources, fostering the integration of interventions into routine practice. We applied a five-phase conceptual framework to evaluate the rigor in the application of process mapping [11]. The five phases are: Phase I—Preparation, Phase II—Data and information gathering, Phase III—Map generation, Phase IV—Process analysis, and Phase V—Take it forward [11]. These five phases were cross-walked with the D&I domains [8]. Because the intervention had already been selected by state agencies, only three D&I domains were involved—adaptation, implementation, and sustainability.

D&I science focuses on understanding how interventions can be disseminated and adopted in real-world settings, while process mapping visually depicts the steps involved in a process. Two types of process maps were generated: a health system-specific map that provided specifics of the process for a participating health system and a high-level map that provided an overview of the process across three participating health systems. On one hand, a health system-specific map can provide detailed insights tailored to an institution and reflect the institution’s unique workflows, challenges, and facilitators. On the other hand, a high-level process map provides a more general overview that can be applied across multiple health systems and used by future systems seeking to implement the program. The high-level map is less detailed, with a broad view of standard workflows and common challenges faced by different organizations.

To create the process maps, we gathered information by reviewing the original EPICC program manual [12], consulting with CommCARE management and PRC representatives, and meeting and communicating with representatives from participating health systems who were involved in the referral process. Based on a five-phase conceptual framework for process mapping [12] and the three D&I domains involved [8], we adapted the criteria to fit the WR EPICC program and used the framework to evaluate the rigor in our application of process mapping (Table 1). A draft high-level map and three draft health system-specific process maps were initially generated by incorporating information from the EPICC program manual [12] and insights from selected collaborators. These draft maps were presented to each health system for review. Feedback was gathered from collaborators and incorporated into the final maps. After confirming the accuracy of each health system’s implementation, the different maps were aggregated to create a high-level process map reflecting the general processes implemented across the participating health systems. The final maps were then validated by collaborators and verified as reflecting their implementation. The initial draft of the high-level map depicts how the implementation was intended, whereas the final high-level map reflects the current practice as implemented [11]. This project was considered as quality improvement by the Institutional Review Boards (IRB) of University of Missouri—Kansas City, University of Kansas Medical Center, and Saint Luke’s Health System, so participants’ consent was not required.

## 3. Results

During the process mapping exercise, the quality improvement team followed the criteria outlined in Table 1. In the adaptation domain, we found that three health systems adhered to the WR EPICC program’s foundational elements while adapting to accommodate the unique needs and circumstances of different healthcare environments. In the implementation domain, we found that it is important to involve PRCs in the process and that getting feedback from collaborators is essential.

Two PRCs were involved in the review of health system-specific process maps. In addition, we met and communicated with representatives (n = 6) from a health system of two hospitals (Health System A, a large urban health system). We also met and communicated with representatives (n = 3) from Health System B (a large urban health system) and representatives (n = 4) from Health System C (a small rural health system). Health system-specific maps were not developed for informal health system partners. We recruited more representatives from Health System A because it had more client referrals to PRCs. Representatives of health systems (n = 13) included ED physicians, social workers, and health system administrators responsible for medical care, emergency services, recovery services, critical care services, research and innovation, quality and risk management, and transitions of care.

The health system-specific maps varied among the three health systems with health system-specific processes leading to the initial PRC contact with clients (Appendix A). For example, in addition to the typical operations involving a referring physician, Health Systems A has non-typical operations involving social workers. Health System B places the WR EPICC program under its initiative for community health needs assessment. Health System C has community partners or outreach programs, including the local police department, to support the WR EPICC program. As such, Health Systems B and C have made engagement efforts to establish a presence within the community.

**Figure 1:**
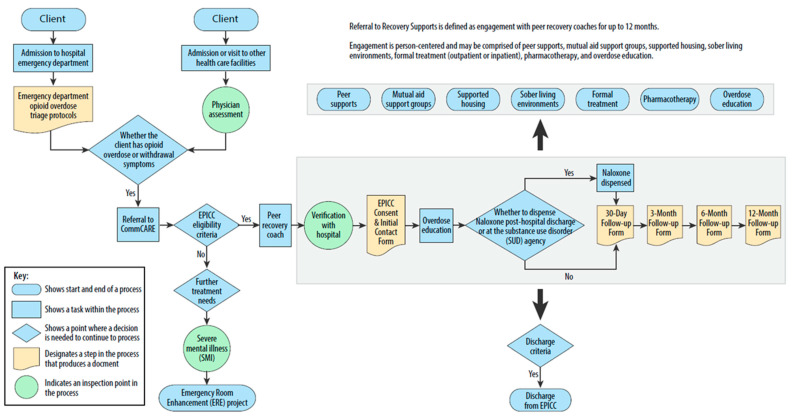
High-level process map of the WR EPICC program.

The high-level map summarized the practice patterns across the health systems. It identifies two client entry points: evaluation in the ED, or admission or visit to other health care facilities (Figure 1). To enroll a client in the WR EPICC program, a PRC verifies client information with the health system, and the client completes the EPICC Consent and the Initial Contact Form. In addition to overdose education, naloxone may be dispensed after discharge or at a substance use disorder agency. The PRC follows up with the client at one month, three months, six months, and 12 months, represented by the large rectangle area. During the initial weeks, the PRC frequently contacts the client. As the client becomes involved with other services later, the PRC reduces the frequency of contact, typically checking in every few weeks. Furthermore, referral to recovery supports is provided for up to 12 months, including peer supports, mutual aid support groups, supported housing, sober living environments, formal treatment, pharmacotherapy, and overdose education, represented by the small rectangle area. Overall, the high-level process map depicts a recovery ecosystem that includes the essential steps of referral to the WR EPICC program, initiation of recovery, referral from the WR EPICC program to recovery supports, maintenance of recovery, and overdose prevention.

## 4. Discussion

While there are multiple possible strategies for addressing the opioid epidemic, developing a community-based infrastructure that could be leveraged across multiple health systems is a potentially efficient strategy for regional efforts to improve opioid use disorder treatment. To better understand a regional strategy, the WR EPICC program, we formally examined the processes of its implementation by different regional health systems. Comparing health system-specific process maps allows for a comprehensive examination of how implementation strategies varied across different contexts. This analytical approach involved scrutinizing steps in the process maps to discern variations, which is crucial for identifying context-specific modifications. While we identified variability across three health systems, primarily in the processes leading to the initial PRC contact with clients, there were also commonalities across all implementation efforts. These included similar client entry points and consistent PRC engagement with clients. In terms of RPC engagement, a PRC meets with a client who is eligible and verifies the client’s information with the health system. The client completes the consent and initial contact form. The client is then officially enrolled in the WR EPICC program. Collectively, we believe that clearly understanding the key components of implementing the WR EPICC program for clients with opioid use disorder can enable future health systems to more efficiently implement the program.

Our study extends the growing literature on the value of process mapping to offer a visual representation of processes for understanding and guiding implementation efforts. Antonacci et al. reported that five factors were associated with successful process mapping, including information from multiple collaborators, facilitator experience and skills, training, and iterative use of process mapping [9]. We considered these factors when evaluating the rigor in the application of process mapping (Table 1). In addition, using these criteria sets this project apart from many of the studies that used process mapping. There is a large variation in how existing studies of process mapping meet the criteria of the five-phase conceptual framework; for example, only 15% of the studies meet the criterion “the team is educated/trained on the use of process mapping tool” and 19% meet the criterion that “sticky notes or paper-based maps are transferred to the charting software as soon as possible” [11]. By using the criteria outlined for successful process mapping, the insights from this evaluation of the WR EPICC program should be generalizable and support future implementation efforts. Moreover, the final high-level process map incorporated empirical data and observations from actual practice, making it a more accurate representation of lived experiences than the draft high-level map which was constructed from the EPICC program manual. This realism could enhance its applicability and reliability in guiding future implementations. Transforming the draft into the final high-level map was a collaborative effort, with input and experiences of collaborators that were crucial in shaping the final high-level map and ensuring its accuracy, comprehensiveness, and relevance. The iterative refinement from the draft to the final high-level map [11] embodies the principles of continuous improvement.

It is important to distinguish between draft maps and the final maps. The draft maps represent the ideal description of how the implementation should be done, typically created based on theoretical models, best practices, and aspirational goals. For example, the draft high-level map in this study was created based on the EPICC program manual [12], and it served as a blueprint to guide the implementation process. In contrast, the final high-level map reflected current practice as the WR EPICC program was being implemented. The final maps were different from the draft maps because they incorporated the realities and complexities encountered during each hospital’s implementation. These could include unanticipated challenges, resource constraints, and technical issues, resulting in deviations from the original plan. The final maps were representations of how the process actually worked, including health systems’ workarounds and adjustments. As such, the final high-level map provides a practical, realistic view of the implementation process and insight into which aspects of the original plan were achievable and which were not. By comparing the draft and final maps, researchers and collaborators can identify areas for improvement, understand the impact of various constraints, and improve implementation strategies.

The availability of a rigorously-developed process map can support three of the five D&I domains (Table 1). In the adaptation domain, careful consideration must be given to balancing implementation fidelity to the core components of the WR EPICC program and contextual factors while allowing for necessary adaptations within a health system. Implementation fidelity is the extent to which programs are implemented as intended [13] and acts as a potential moderator between interventions and intended outcomes [14]. We found referral to recovery supports, including peer supports, mutual aid support groups, housing, sober living environments, formal treatment, pharmacotherapy, and overdose education, is a CommCARE practice and a contextual factor, but it was not described in the EPICC program manual [12]. The adaptation domain was found to be relevant to all five phases of process mapping, indicating that for the WR EPPIC program, the administration of a health system should expect a significant investment of time in adapting the EPICC program manual [12] to fit the health system. All five phases were identified to be relevant to the implementation domain, again indicating to health systems that to implement the WR EPICC program, there must be a presence of all collaborators from all parts of the program in all phases. Finally, the sustainability domain was found to be relevant to two phases, “process analysis” and “take it forward”. For the WR EPICC program to be sustainable, several factors are important, including development of detailed maps, feedback on the maps for validation, and making appropriate changes to the WR EPICC program based on the knowledge gained from the process mapping.

By cross-walking the EPICC program to the two of the five D&I domains, implementation and sustainability, future health systems can use our results to identify areas for improvement and address challenges. A future research direction could be to use the process maps to evaluate implementation fidelity. There would be five elements to measure: adherence to an intervention, exposure or dose, quality of delivery, participant responsiveness, and program differentiation [13,15,16]. Qualitative data could be collected through interviews with collaborators. Then, for each health system, the interview results could be compared with its detailed process map generated in this project. The future of process mapping and D&I science are also poised for change as technology and interdisciplinary collaboration continue to shape the landscape. Process mapping has evolved into cloud-based digital platforms that enable real-time updates [17], and D&I science has leveraged digital technology to tailor implementation [18].

Combining D&I science and process mapping allows researchers to gain deeper insights into the mechanisms that influence an intervention. First, process mapping can help researchers and practitioners understand the sequential steps involved from the development of an intervention to its adoption and sustained use in practice. By mapping these steps, it becomes easier to identify critical points at which implementation efforts are most likely to succeed or fail. Second, process mapping can facilitate the identification of key stakeholders. For the WR EPICC program, the collaborators play critical roles. Process mapping helps delineate the roles and responsibilities of each collaborator, as well as their interactions and interdependencies, thereby increasing the likelihood of successful implementation. Involving collaborators allowed us to capture a range of perspectives that enriched the process maps. This approach also facilitated buy-in from collaborators, as the process maps reflected their direct contributions. By using this approach, we ensured that the process maps were grounded in practical experience. In addition, integrating process mapping with D&I domains can improve the evaluation of implementation efforts. Process maps can be used to establish baseline measures of current practices, which can then be compared to post-intervention outcomes. This comparison allows researchers to assess the impact of the intervention on various aspects of the implementation process.

Health System B has its initiative for community health needs assessment, and Health System C has community partners or outreach programs. By having representatives attend local health fairs or support group meetings, a health system can interact directly with community members, raise awareness of the WR EPICC program, and build trust and rapport with individuals who may benefit from the WR EPICC program. In addition, by providing educational resources, a health system and the WR EPICC program can empower individuals with the knowledge and skills necessary to achieve and maintain recovery. In fact, the Eastern Region EPICC program has positioned itself as a supportive entity in the community recovery landscape, with the dedicated role of a Community Engagement Specialist [5]. The Community Engagement Specialist has conducted weekly outreach initiatives aimed at bridging gaps and providing essential resources to local agencies and potential clients. Through a regular presence at domestic violence shelters and community centers, the Community Engagement Specialist has provided support in these vulnerable communities. The WR EPICC program may consider adding a similar dedicated community engagement role in the future.

Our work should be interpreted in the context of the following potential limitations. First, process mapping inherently has some limitations, including the oversimplification and static representation of a dynamic process. By attempting to present complex processes in a simplified, linear format, some details may be lost. Processes are often dynamic and subject to change, so a process map created at one point in time can become outdated, making it less useful for continuous improvement efforts. Although these maps were serially updated by researchers and validated by collaborators, keeping process maps current can be a resource-intensive task that requires ongoing attention and adjustments. In addition, process mapping involves interpretation, and different people may perceive and document processes differently, which can introduce bias and inconsistency. Nevertheless, we used the criteria to evaluate the rigor in the application of process mapping to minimize such biases (Table 1). Finally, process mapping should be seen as the first step in a continuous improvement journey, not an end in itself. The usefulness of the health system-specific and high-level maps in understanding health system-level variations in referrals and clients’ long-term outcomes has yet to be investigated.

Future research could explore which type of mapping leads to more improvements in clients’ outcomes and operational efficiency. For instance, studies could compare health system-specific maps and high-level maps in terms of their impact on reducing client wait times, improving care coordination, or increasing client satisfaction. It is also important to consider the adaptability and scalability of process maps, as health systems vary widely in size, scope, and resources. Research could focus on how well different types of process maps can be adapted to the varied sizes and complexities of different health systems.

## 5. Conclusions

In this project, process mapping improved the understanding and implementation of WR EPICC across the D&I domains of adaptation, implementation, and sustainability. Through the use of process mapping, collaborators gained a comprehensive understanding of the relationships within the WR EPICC program, leading to more information that can be considered for ongoing adaption and process improvements.

## Figures and Tables

**Table 1:** Criteria to evaluate rigor in the application of process mapping.

Phase of Process Mapping [11]	Conceptual Framework Criteria [11]	D&I Domain [8]
Phase 1: Preparation, planning, and process identification	Service providers and the client group were clearly identified. The project team learned the process mapping tool. PRC representatives were involved, and they represented the perspectives of PRCs and clients.	Adaptation, implementation
Phase 2: Data and information gathering	Information was gathered to inform the process mapping exercise.	Adaptation, implementation
Phase 3: Map generation	Different perspectives from collaborators were gathered.	Adaptation, implementation
Phase 4: Process analysis	The draft high-level map and health system-specific maps were reviewed by the project team. Feedback from collaborators was gathered during the process mapping exercise, and analysis and feedback were represented on the final maps. Notes or paper-based maps were transferred to the charting software timely. The final maps were validated by collaborators.	Adaptation, implementation, sustainability
Phase 5: Take it forward	Further actions based on knowledge gained from process mapping were undertaken, demonstrating implementation.	Adaptation, implementation, sustainability

**Figure A1:**
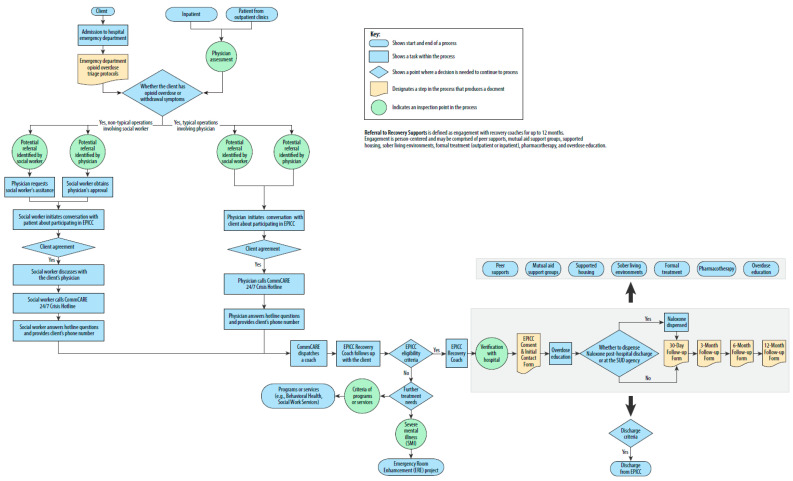
Health System A-specific process map.

**Figure A2:**
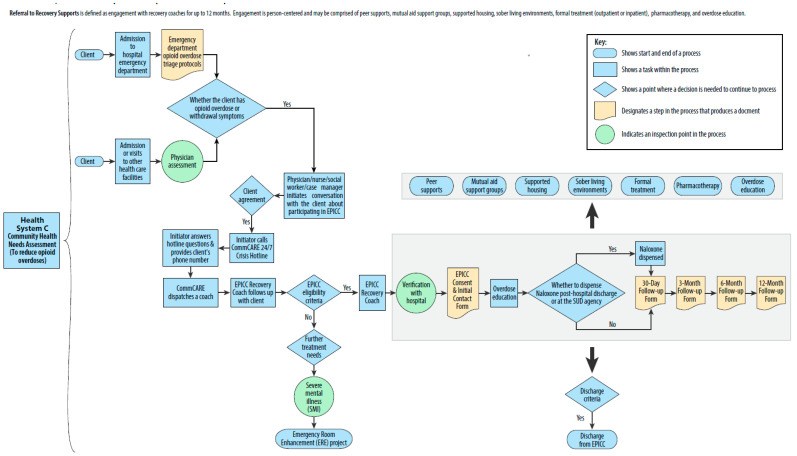
Health System B-specific process map.

**Figure A3:**
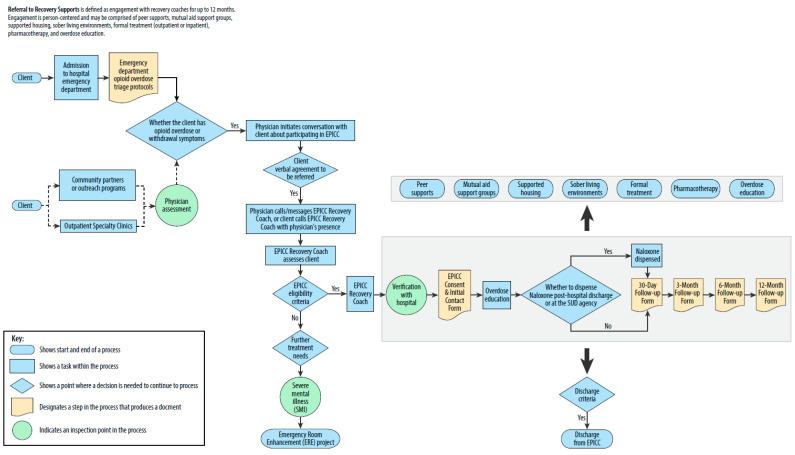
Health System C-specific process map.

## Data Availability

Data are contained within the article.
